# Biological causes of immunogenic cancer cell death (ICD) and anti-tumor therapy; Combination of Oncolytic virus-based immunotherapy and CAR T-cell therapy for ICD induction

**DOI:** 10.1186/s12935-022-02585-z

**Published:** 2022-04-29

**Authors:** Amirhossein Mardi, Anastasia V. Shirokova, Rebar N. Mohammed, Ali Keshavarz, Angelina O. Zekiy, Lakshmi Thangavelu, Talar Ahmad Merza Mohamad, Faroogh Marofi, Navid Shomali, Amir Zamani, Morteza Akbari

**Affiliations:** 1 Department of Immunology, School of Medicine, Shahid Beheshti University of Medical Sciences, Tehran, Iran; 2 Department of Prosthetic Dentistry, I. M. Sechenov First Moscow State Medical University (Sechenov University), Moscow, Russia; 3 Medical Laboratory Analysis Department, College of Health Science, Cihan University of Sulaimaniya, Suleimanyah, Kurdistan region Iraq; 4 College of. Veterinary Medicine, University of Sulaimani, Suleimanyah, Iraq; 5 Department of Hematology and Blood Banking, School of Allied Medical Sciences, Shahid Beheshti University of Medical Sciences, Tehran, Iran; 6 Department of Pharmacology, Saveetha Dental College, Saveetha Institute of Medical and Technical Science, Saveetha University, Chennai, India; 7 Department of Pharmacology and Toxicology, Clinical Pharmacy, Hawler Medical University, College of Pharmacy, Kurdistan Region-Erbil, Iraq; 8 Immunology Research Center, Tabriz University of Medical Sciences, Tabriz, Iran; 9 Department of Immunology, Faculty of Medicine, Tabriz University of Medical Sciences, Tabriz, Iran; 10 Shiraz Transplant Center, Abu Ali Sina Hospital, Shiraz University of Medical Sciences, Shiraz, Iran; 11 Department of Medical Biotechnology, Faculty of Advanced Medical Sciences, Tabriz University of Medical Sciences, Tabriz, Iran

**Keywords:** Oncolytic virus (OV), Chimeric antigen receptor (CAR) T-cell therapy, Immunogenic cancer cell death (ICD), Immunotherapy

## Abstract

Chimeric antigen receptor (CAR) T-cell therapy is a promising and rapidly expanding therapeutic option for a wide range of human malignancies. Despite the ongoing progress of CAR T-cell therapy in hematologic malignancies, the application of this therapeutic strategy in solid tumors has encountered several challenges due to antigen heterogeneity, suboptimal CAR T-cell trafficking, and the immunosuppressive features of the tumor microenvironment (TME). Oncolytic virotherapy is a novel cancer therapy that employs competent or genetically modified oncolytic viruses (OVs) to preferentially proliferate in tumor cells. OVs in combination with CAR T-cells are promising candidates for overcoming the current drawbacks of CAR T-cell application in tumors through triggering immunogenic cell death (ICD) in cancer cells. ICD is a type of cellular death in which danger-associated molecular patterns (DAMPs) and tumor-specific antigens are released, leading to the stimulation of potent anti-cancer immunity. In the present review, we discuss the biological causes of ICD, different types of ICD, and the synergistic combination of OVs and CAR T-cells to reach potent tumor-specific immunity.

## Introduction

Cancer therapies nowadays concentrate on triggering controlled immunogenic apoptosis in tumor cells. However, systemic treatment-related toxicity is still a significant restriction in chemotherapy. Oncolytic virotherapy has emerged as a new cancer treatment strategy that addresses drug accessibility and chemotherapy side effects [1]. Oncolytic viruses (OVs) enter tumor cells preferentially, proliferate, and ultimately induce cell lysis, unleashing additional synthesized viruses, which target and destroy neighboring cells [2]. Likewise, OVs potentially stimulate adaptive immunity against infected tumor cells by lysis of tumor cells and subsequent release of tumor-associated antigens (TAAs), damage-associated molecular patterns (DAMPs), and pathogen-associated molecular patterns (PAMPs). These processes result in the stimulation of antigen-presenting cells (APCs) and the priming of anti-tumor adaptive immune responses [3].

Immunogenic cell death (ICD) offers a potential approach to improving the efficacy of cancer therapy. It is a functionally distinct response pathway that involves the development of cellular stress, followed by cell death and the release of a variety of DAMPs [4]. In fact, ICD promotes the development of innate and adaptive immune responses by increasing adjuvanticity via DAMP release and antigenicity through APC recruitment [5]. Treatment-driven ICD has been shown to induce anti-tumor immune responses that enhance the therapeutic advantages of conventional anti-tumor radiotherapies and chemotherapies [6–8]. There are several ICD inducers, such as radiation, anthracycline chemotherapeutics, and high hydrostatic pressure; alongside these, OVs have developed as a new class of therapeutic approaches capable of inducing ICD [9–11]. CAR T-cell therapy is a type of cellular therapy in which a patient's T lymphocytes are redirected to precisely target and destroy cancer cells [12].

Monotherapy methods for cancer therapy are likely to fail because of inter- and intra-patient heterogeneity of cancer, the diverse nature of cancer cells genomes, and the dynamic condition of the tumor milieu. A synergy between OVs and CAR T-cells seems to be the ideal way to organize a multi-pronged attack on several fronts against frequently quickly developing targets [13]. OVs have the ability to operate in combination with CAR T-cells, assisting them in overcoming some of the many hurdles encountered in solid tumors. First, OVs can lead to the release of danger signals through ICD that may reverse tumor immunosuppression, enabling expansion, activation, and recruitment of CAR T-cells in the tumor microenvironment (TME) [14]. Second, the selective directed lytic function of OVs on tumor cells causes lysis of infected tumor cells and subsequent TAAs release, which may trigger a tumor-specific immune response that has the potential to prevent tumor escape due to loss of antigen or antigen heterogeneity. Third, therapeutic transgenes may be inserted into OVs, potentially enhancing the effector capabilities of T cells [15].

Here, we provide a review of the biological causes of ICD and its potential role in the induction of anti-tumor immunity through OVs replication within cancer cells, and also the synergistic combination of OVs with CAR T-cell to achieve potent tumor-specific immunity.

## Cell death pathways in cancer

Cell death is an essential process in biological activities and plays an important role in homeostatic equilibrium [16]. Mammalian tumors gradually lose their ability to launch apoptotic cell death processes, making them resistant to apoptosis-targeting chemotherapeutic treatments [17]. As a result, alternative cell death mechanisms must be discovered in order to develop effective cancer therapies.

Apoptosis, pyroptosis, necroptosis, ferroptosis, and autophagy-dependent cell death are among the cell death pathways identified thus far, all of which are categorized as ICD [18–20]. Apoptosis is a kind of non-inflammatory programmed cell death that may be triggered by either intrinsic or extrinsic stimuli. Apoptosis, mediated by caspase-2, -3, -6, -7, -8, and -9, is involved in a number of pathological conditions, notably cancer [21, 22]. The intrinsic apoptosis pathway, which involves mitochondria, is activated by a variety of microenvironmental stimuli, including loss of growth factor signaling or fatal events within the cell, such as DNA damage, reactive oxygen species (ROS) excess, hypoxia, or chemotherapeutic drugs [18, 23–25]. The extrinsic apoptosis pathway, on the other hand, is triggered as specific ligands released by other cells stimulate the transmembrane death receptors. Tumor necrosis factor (TNF) is a class of proteins that includes death receptors. TNF receptors also contain a cysteine-rich extracellular subdomain that enables them to identify their ligands precisely, as well as a cytoplasmic domain termed as the "death domain (DD)" that is responsible for conveying the death signal from the cell's surface to intracellular pathways [26]. Since there is no loss of membrane integrity, apoptosis is typically thought to be a non-immunogenic type of cell death that prevents the leakage of intracellular contents. On the other hand, apoptosis has recently been discovered to be immunogenic, through the release of DAMPs, under stress situations like chemotherapies or physical modalities [27].

Pyroptosis is a kind of programmed cell death that often takes place in response to intracellular pathogen infection. It is characterized by swelling of cell and plasma membrane disruption, allowing cytosolic contents to escape into the extracellular environment. However, it is increasingly being studied as a potential cell death mechanism in cancer therapy. Gasdermins, inflammasomes, and pro-inflammatory cytokines are all essential components of pyroptotic cell death pathways and have been implicated in the onset and development of cancer. Interfering with these pathways might be a promising therapeutic option for cancer treatment [28, 29]. Pyroptosis might be induced by two different inflammasome pathways: canonical and non-canonical. Caspase-1 is responsible for canonical pyroptosis, which is activated by a variety of PAMPs and DAMPs, while non-canonical pyroptosis is triggered by intracellular lipopolysaccharide (LPS) and involves human caspase-4/-5 [30–32]. Inflammation-induced tumor development may originate from Caspase-1 deficiency [29]. Cell swelling and plasma membrane rupture characterize necrosis, which is commonly induced by major chemical or physical stressors such as the presence of toxins or trauma [33]. Different types of cell death have been discussed in Table 1.

**Table1:** Comparison of multiple forms of cell death

	Intrinsic apoptosis	Extrinsic apoptosis	Necrosis	Necroptosis	Ferroptosis	Pyroptosis	Refs.
Initiators	Loss of growth factor signals, DNA damage, reactive oxygen species (ROS) excess, hypoxia, chemotherapeutic medicines	Extracellular microenvironment, TNF-*α*, FasL (death ligand binds its receptor), and infectious pathogens	Toxins, infections, trauma	Ischemic injury, FASL, TNFR1	Erastin, RSL3, iron accumulation and lipid peroxidation,	LPS, DAMPs, microbial infections	[18, 23–25, 34, 41, 190–192]
Intermediate signalings	Mitochondrial pathway Caspase-3, -6, -7, -9-dependent	Caspase-3, -8-dependent	Danger signals, ROS	Caspase-independent RIP1/RIP3/MLKL necrosome	Iron-dependent production of ROS	Nod-like receptors (NLRP3) Caspase 1-dependent pyroptosome, caspase-4/5/11	[193]
Morphological features	Retention of plasma membrane integrity, cell shrinkage, DNA fragmentation, phosphatidylserine (PS) exposure, and the formation of apoptotic bodies	Retention of plasma membrane integrity, cell shrinkage, DNA fragmentation, phosphatidylserine (PS) exposure, and the formation of apoptotic bodies	Loss of plasma membrane integrity, cell swelling, leak of content	Membrane permeabilization, swollen cellular organelles	Cytoplasmic swelling (oncosis), chromatin condensation, swelling of cytoplasmic organelles and loss of membrane integrity	Pore formation, swelling of cell, rapid loss of plasma membrane	[56, 194–198]
DAMPs released	CRT, ATP (at pre-apoptotic stage), HMGB1(at late-apoptotic stage), histones, Annexin A1 (ANXA1)	CRT, ATP, HMGB1, histones (release of nuclear DAMPs following DNA fragmentation), Annexin A1 (ANXA1)	HMGB1, ATP, histones, HSPs	ATP, CRT,	HMGB1, DNA, lipid oxidation products such as 4HNE, LTB4, LTC4, LTD4 and PGE2	ATP, HMGB1	[199–201]
Inflammation	Non-inflammatory	Non-inflammatory	Pro-inflammatory	Pro-inflammatory	Pro-inflammatory	Pro-inflammatory	
Immunogenicity	Non-immunogenic (may be immunogenic under stress situations like chemotherapy or physical modalities)	Non-immunogenic (may be immunogenic under stress situations like chemotherapy or physical modalities)	Very high	High	High	High	[27]

Necroptosis is a kind of necrosis that is characterized by caspase-independent cell death and, unlike apoptosis, induces inflammation through the release of DAMPs [34]. It is primarily triggered by receptor-interacting protein 1 (RIP1), RIP3, and mixed lineage kinase domain-like (MLKL) protein [35]. New evidence shows that necroptosis has pro- or anti-tumoral effects on cancer growth and progression. Necroptosis induction in tumor cells has been investigated as a possible cancer treatment approach [34]. Cancer cell necroptosis is thought to be an ICD that triggers anti-tumor immunity. Although increased necroptosis leads to cancer cells death, excessive cell death also raises the likelihood of surviving cells proliferating and metastasizing by promoting the production of ROS, inflammation, and immune suppression [36–38]. Necroptosis also promotes myeloid cell-induced adaptive immune inhibition, which leads to cancer development. As a result, the overall effect of necroptosis on cancer cells has remained elusive.

Ferroptosis is a newly found form of controlled cell death defined by the accumulation of lipid ROS to lethal levels in the presence of iron [39]. Unlike necroptosis and apoptosis, ferroptosis is independent of receptor-interacting protein 1 kinase (RIPK1) and caspase activity [40]. Ferroptosis was first characterized in 2012 as a distinct process from apoptosis, necrosis, and autophagy. However, recent findings have described ferroptosis as a form of autophagy-dependent cell death [41]. High-mobility group box 1 (HMGB1) is released by cancer cells during ferroptosis in an autophagy-dependent mechanism [42]. It is a critical protein needed for cancer cell immunogenicity as a major DAMP [43].

Interestingly, it has been hypothesized that cancer cells that have escaped conventional types of cell death mechanism may preserve or acquire ferroptosis sensitivity Emerging evidence suggests that triggering ferroptosis might be leveraged to treat cancer, particularly aggressive tumors that are resistant to conventional therapies [40]. Many recent studies in this area have concentrated on designing and developing anti-cancer drugs based on ferroptosis induction [44]. Even in chemo-resistant cancers, strategies that manipulate ferroptosis induction have been shown to successfully suppress tumor growth [45]. In this regard, Wang et al. showed that CD8^+^ T lymphocytes cause ferroptosis in tumor cells in vivo, providing the first direct proof of a link between anti-tumor immunity and ferroptosis [46].

As a result, most cancers have an inherent resistance to apoptosis, thus, inducing cell death pathways other than apoptosis, like necroptosis, pyroptosis, and ferroptosis, has turned into a noteworthy cancer therapeutic method. Moreover, the combination of other immunotherapy approaches, such as immune checkpoint inhibitors (ICIs), with stimulation of necroptosis, pyroptosis, and ferroptosis, have been demonstrated to synergistically increase anti-cancer efficacy [47].

## Immunogenic cell death pathway

ICD is a new concept in tumor cell death that involves both innate and adaptive immune responses and gives an enhanced immunogenic anti-cancer effect to cytotoxic medicines [48, 49]. So far, only a few cytotoxic drugs have been shown to stimulate anti-cancer immunity via triggering ICD [50]. ICD is characterized by changes in the cell surface composition as well as the leak of signaling molecules. These signals enhance the tumor antigen presentation to T lymphocytes by triggering a set of receptors expressed by dendritic cells (DCs). In fact, ICD is a key mechanism for stimulating the immune cells against tumor cells [48].

Dying cells release chemicals that the immune cells may exploit as adjuvants or danger signals. DAMPs are the general name for these signals which are the chemicals that mediate immunogenicity and adjuvanticity of dying cells and are essential for ICD's 'anti-cancer vaccination effect' [51–53]. Interestingly, anti-cancer vaccination effects have been observed in vivo after injection of dying cancer cells subcutaneously undergoing ICD [54]. Pattern recognition receptors (PRRs) on DCs, such as Toll‐like receptors (TLRs) and NOD‐like receptors (NLRs), recognize DAMPs, which then stimulate tumor‐specific immune responses [55, 56]. DAMPs released during ICD comprise chaperones of endoplasmic reticulum (ER) such as heat-shock proteins (HSPs) and calreticulin (CALR) [57], type I interferons (I-IFNs), non-histone chromatin-binding protein HMGB1 [58, 59], ATP [60], annexin A1 (ANXA1) [61], and cancer cell-derived nucleic acids [62]. DAMPs recruit ligands on DCs and trigger DC maturation, which enhances the antigen uptake by DCs. Subsequently, by presenting antigens, DCs trigger T cell-specific responses that eliminate further tumor cells. In the context of strong stimulation of the anti-tumor immune responses, DAMPs enable tumor antigens to be cross-presented to CD8^+^ T cells [63, 64]. Finally, ICD induction leads to long-term immunity against tumor cells [65].

## Multiple cell death pathways initiated by OVs

OVs destroy cancer cells by inducing a variety of cell death pathways. Apoptosis, necroptosis, autophagic cell death, and pyroptosis are among them, each of which serves as the primary death form for a specific OV. OV-induced cancer cell death is primarily immunogenic and has the potential to elicit anti-tumor immune responses [10]. OVs are a category of biological agents that have the potential to treat cancer. This approach has been used in a number of clinical studies that are now underway or have recently been completed. In 2015, talimogene laherparepvec became the first OV to receive the food and drug administration (FDA) approval in the United States, marking a breakthrough in the setting [66, 67].

### OV-mediated induction of cell lysis

**Fig. 1:**
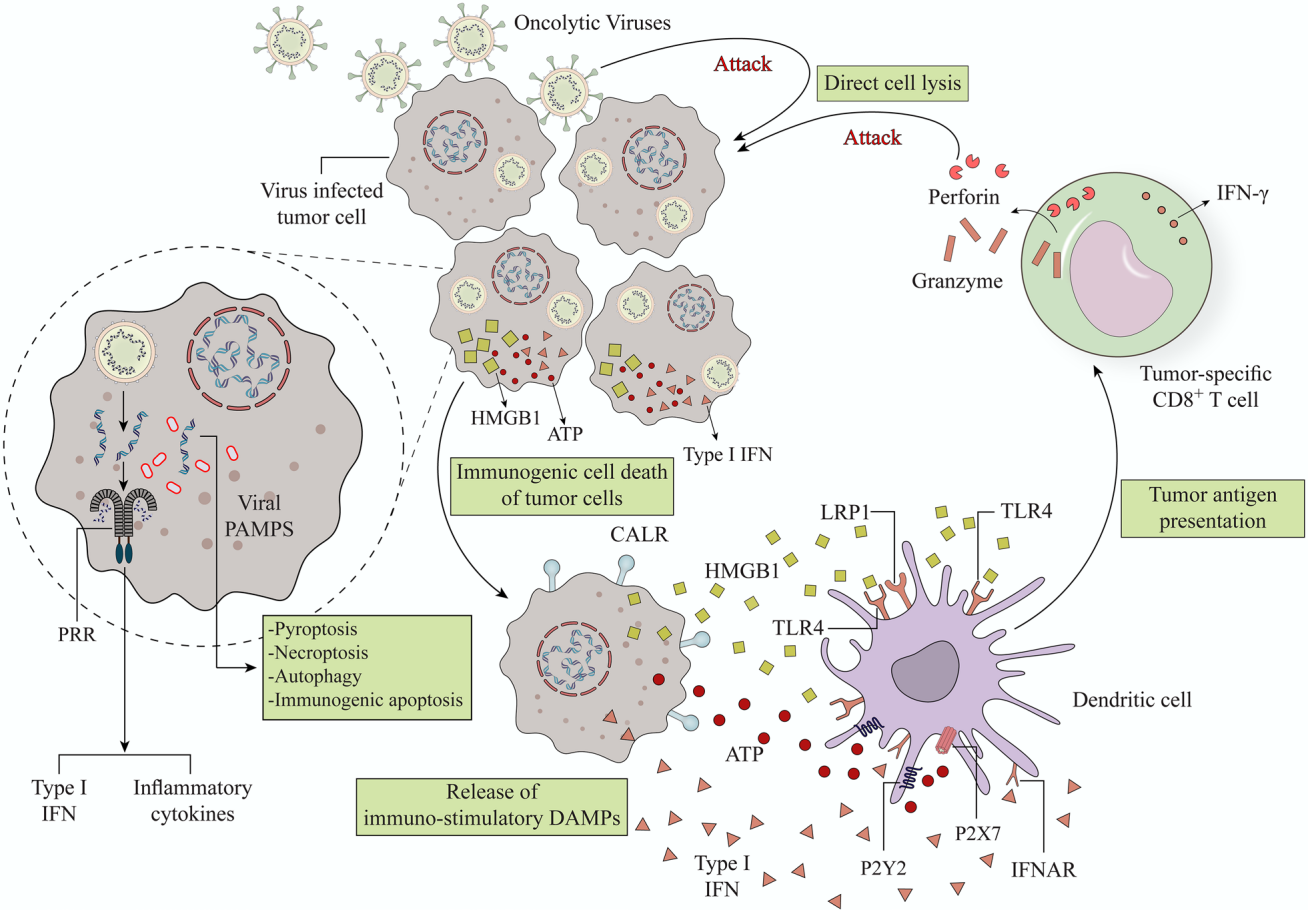
Mechanism of immunogenic cell death induction via oncolytic viruses and priming of anti-tumor specific responses mediated by antigen presenting cells. Oncolytic viruses (OVs) attack and destroy tumor cells preferentially. Lysis of tumor cells releases TAAs and PAMPs which trigger PRRs, which then produce inflammatory cytokines and antiviral type I IFNs. Viruses can activate cell death pathways, resulting in immunogenic cell death phenotypes such as necroptosis, pyroptosis, immunogenic apoptosis, and autophagic cell death. Subsequently DAMPs such as ATP, HMGB1, CALR, and type I IFNs are released by ICD from dying cancer cells. Antigen-presenting cells, such as DCs, are recruited to the tumor site. P2Y2 and P2X7 are purigenic receptors that increase DC recruitment and maturation, respectively, when extracellular ATP binds to them. CALR enhances phagocytosis and the production of proinflammatory cytokines through binding to LRP1. Also, binding HMGB1 to TLR-4, promote cytokine production and cross-presentation of antigen. IFNs bind to IFNR and promote the production of a vast number of IFN-stimulated genes that help to induce adaptive immune responses. Mature DCs can present cancer-related Ags to cancer-specific T cells, resulting in anti-tumor immunity and cytolysis mediated by perforin and granzyme B. *HMGB1* High mobility group box 1, *ATP* adenosine triphosphate, type-I *IFN* type-I interferon, *CALR* calreticulin, *PRR* Pattern Recognition Receptor, *TLR4* Toll-like receptor 4, *LRP1* low density lipoprotein receptor–related protein 1, *IFNAR* interferon-α/β receptor, *DAMPs* Damage-associated molecular patterns, *ICD* Immunogenic cell death, *TAAs* tumor-associated antigens, *PAMPs* Pathogen-Associated Molecular Pattern, *DCs* Dendritic cells

OVs provide an interesting therapeutic combination of cancer cell lysis and immune activation, making them promising in situ cancer vaccines as well as they are easy to combine with other drugs [67]. The release of cellular debris and viral antigens in the TME stimulates the immune responses [67] (Fig. 1). OVs induce oncolysis, which is followed by the production of infectious viral progeny that spreads to surrounding tumor cells, as well as subproducts such as viral particles, PAMPs, DAMPs, tumor cell debris, and TAAs. All of these activities contribute to the local and systemic stimulation of innate and adaptive anti-cancer immune responses [9, 68, 69]. Because OVs predominantly replicate in tumor cells, they can be designed to express transgenes that enhance their immuno-stimulatory capabilities, as well as to regulate the TME to improve the eradication of tumors by the immune cells [70]. Various kinds of OVs have been employed as natural or manufactured vectors for the treatment of cancer, such as vaccinia viruses (VVs), adenoviruses (Ads), measles viruses (MVs), herpes simplex viruses (HSVs), vesicular stomatitis viruses (VSVs), Coxsackie viruses, Seneca Valley viruses (SVVs), Newcastle disease viruses (NDVs), Myxoma viruses (MYXVs), polioviruses, parvoviruses, and retroviruses [71].

Deficiency in innate immunity potentially enables OVs to infect and propagate [72]. Antiviral I-IFN signaling is defective in many cancer cells but not in healthy ones, allowing for selective virus replication [73]. The concurrence of viral infection and cell lysis, which triggers the release of tumor antigens and DAMPs, may overcome the immunosuppressive features of the TME and enhance anti-cancer immunity [67]. APCs release cytokines as virus replication and tumor cell lysis progress, gradually attracting other adaptive immune cells. The eventual aim of this immune stimulation procedure is to prime T cells toward targeted tumor antigens so that adaptive immunity can be established [74]. Multiple clinical trials have demonstrated evidence of OV-induced anti-tumor immune responses. For example, patients diagnosed with melanoma who treated Talimogene laherparepvec (T-VEC) (an example of Herpesviruses) or coxsackievirus (an example of Picornaviruses) in separate clinical trials had a higher amount of CD4^+^ and CD8^+^ T cells than untreated ones [75–77].

Most OVs induce cell lysis in a variety of ways throughout their entire life cycle. Retroviruses, for example, have the potential to be beneficial agents since they easily infect mitotic cells and disseminate rapidly, however they do not necessarily lead to cell lysis [78].

### OV-mediated induction of autophagy and necroptosis

OVs have been found to disrupt the intracellular mechanism of autophagy [79]. Moreover, autophagy has been shown in many cancer models to either promote carcinogenesis or function as a tumor suppressor [80]. Autophagy is a catabolic process that generates energy through the lysosomal breakdown of intracellular components in response to various stimuli, including hypoxia, nutrient shortage, and infection [81]. Through promoting oncolysis and ICD, autophagy can boost replication and infectivity of the OVs and improve their anti-tumor effects [71]. For example, oncolytic paramyxoviruses have been discovered to trigger autophagy and cause tumor cell death. Indeed, autophagy induction has been shown to improve tumor cell immunogenicity by releasing DAMPs and TAA and activating autophagy-related ICD. TAAs are then cross-presented to CD8^+^ T lymphocytes via the major histocompatibility complex class I (MHC-I), resulting in effective priming of the immune response against tumor [82]. However, some research showed that autophagy can decrease the cytotoxicity and anti-tumor activity of OVs via supplying tumor cells with survival resources [71]. OVs induce autophagy, which suppresses anti-virus innate immune responses, allowing viruses to replicate more rapidly. While I-IFN signaling deficiency in cancer cells is a positive factor for OV replication within tumor cells, there is data that several cancer cells maintain the I-IFN responses that drive resistance of tumor cells against oncolytic virotherapy [83]. Nevertheless, other research suggests that oncolytic Ad-induced autophagy may have no impact on viral replication in infected cancer cells. In this regard, Yokoyama et al. showed that OBP-405, an oncolytic Ad, has a significant anti-cancer impact on glioblastoma cells. Additionally, the cytotoxicity of OBP-405 was diminished following pharmacological inhibition of autophagy [84]. Autophagy not only plays an important function in infectivity and replication of the OVs, but it also plays a role in oncolytic virotherapy by mediating ICD [85]. Liikanen et al., for instance, reported that combining oncolytic Ads 5/3-D24-GM-CSF with temozolomide (TMZ) reduced tumor development, promoted autophagy, and triggered ICD through increasing ATP secretion, CALR, and HMGB1 expression [86]. Also, OBP-301 was shown to cause autophagic cell death via the E2F1‐microRNA‐7‐epidermal growth factor receptor (E2F1-miR7-EGFR) pathway. Mechanistically, MiR-7 upregulation is induced by increasing E2F1 expression and suppressing oncogenic EGFR expression, which reduces cell survival and promotes autophagy [87]. Numerous OVs have been demonstrated to regulate autophagy to activate both innate and adaptive immune responses through promoting antigen presentation and cytokine production [3]. Table 2 summarizes recent advancements in oncolytic immunotherapy employing OV-mediated autophagy.

**Table2:** Oncolytic Virus (OV)-modulated autophagy in oncolytic-based immunotherapy

Oncolytic Virus (OV)	Effect of OV on Autophagy	Effect of Autophagy on oncolytic-based immunotherapy	Refs.
OBP‐301	Induces autophagic cell death through an E2F1-miR-7-EGFR pathway	Provides information on oncolytic virotherapy's anticancer mechanism	[87]
OBP-702: p53-armed hTERT-Ad	Induces apoptosis and autophagy	Induces anti-tumor immune responses through regulation of miRNA and DRAM in human osteosarcoma cells	[202]
Oncolytic adenovirus OBP-405	Enhances of autophagic pathway	Anti-tumor effects on glioblastoma cells through combining autophagy-inducing agents, such as temozolomide (TMZ) and rapamycin	[84]
Ad (OBP-301)	Induces autophagy-associated cell death	Induces anti-tumor immune responses through production of DAMPs, such as uric acid, which promotes innate immune cells to produce interleukin 12 (IL-12) and interferon-γ (IFN-γ)	[203]
Ad(Δ24FvIII)	Induces autophagy by activation of c-Jun N-terminal kinase (JNK) signal transduction pathway	Adenoviruses and autophagy inducers in combination may improve the processing and presentation of cancer-specific antigens integrated into capsid proteins	[204]
Adenoviruses 5/3-D24-GM-CSF	Induces tumor cell autophagy	Combines oncolytic Ad 5/3-D24-GM-CSF with temozolomide (TMZ) and metronomic cyclophosphamide (CP) trigger immunogenic cell death and anti-tumor immune responses through increasing ATP secretion, calreticulin (CRT), and high-mobility group box-1 expression (HMGB1)	[86]
Herpes simplex virus type 2 (ΔPK)	Induces autophagy	Increases the release of inflammatory cytokines granulocyte macrophage colony-stimulating factor, TNF-α, and IL-1β via autophagy-mediated stimulation of Toll-like receptor 2 pathways and pyroptosis	[205]
Newcastle disease virus (NDV), strain FMW (NDV/FMW)	Induces apoptosis and/or autophagy in cancer cells	Induces NDV-mediated immunogenic cell death (ICD) in lung cancer cells	[206]
Hitchner B1 (HB1) strain of newcastle disease virus	Induces autophagy in TC-1 cell line in a dose-dependent manner	Introduces HB1 NDV as a powerful candidate for the cervical cancer therapy	[207]
Newcastle disease virus (NDV)	Induces autophagy in ICD	Induces ICD in tumor cells, which primes adaptive immunity against tumor	[89]
Newcastle disease virus (NDV) strain NDV/FMW	NDV/FMW triggers autophagy in A549/PTX cells via dampening the class I PI3K/Akt/mTOR/p70S6K pathway, which inhibits autophagy	Combines NDV/FMW with autophagy modulators is an effective way to boost NDV/FMW therapeutic function in drug-resistant lung malignancies	[208]
AdΔ24	Induces autophagy act as cytoprotective function	Autophagy might play a survival function in AdΔ24-infected ovarian cancer cells	[209]
Telomelysin (Ad)	Induces autophagic cell death	Enhances the synthesis of inflammatory cytokines like IL-1, TNF-, and IL-6, as well as neutrophil chemotactic factors like IL-8/CXCL8 and S100A8/A9	[203, 210]

Jing Ma et al. showed that Ad, SFV, and VV could induce various ICD while also stimulating anti-tumor immune responses [88]. They demonstrated that autophagy is typically activated by the Ad-infection of cancer cells. Moreover, Ad stimulates necroptotic and pyroptotic cell death processes. In contrast, SFV infection mainly triggers immunogenic apoptosis, whereas VV infection induces necroptosis [88]. Previous research has shown that autophagy activation by an oncolytic virus infection, as well as the viruses' effects on autophagy, are complicated and virus-specific [71].

The biological response of glioblastoma cells to NDV infection has recently been recognized as necroptosis [89]. TNF members, TLRs, and DNA and RNA sensors can all trigger necroptosis, which is a type of programmed cell death that is not dependent on caspase 8. The receptor-interacting protein kinase 1 (RIPK1)-RIPK3 complex is required for signal transduction, and Necrostatin-1 inhibits this complex [90].

In another study, Chen et al. demonstrated that stereotactic body radiotherapy (SBRT) in combination with oncolytic VV can induce necroptosis of tumor cells and activate macrophages via the production of DAMPs, resulting in significant anti-tumor immunity. As a result, combination therapy has the potential to be widely applied in clinical cancer treatment [91].

### OV-mediated induction of apoptosis

OVs can preferentially propagate in tumor cells and trigger apoptosis without destroying healthy tissues, making them a hopeful approach in cancer treatment [92]. OVs might be equipped with pro-apoptotic genes, which are commonly lost in cancer [93]. Several studies reported that OVs could induce apoptosis, For example, Washburn et al. revealed that NDV stimulates apoptosis in cancer cells and directly provokes the innate immune system via enhanced cytokine production like type I IFN, RANTES (CCL5), GM-CSF, and IL-12 and enhanced antigen presentation [94]. VSV stimulates apoptosis in tumor cells rapidly and efficiently, which is the foundation for its oncolytic capability [1]. Also, Miyagawa et al. demonstrated that the urokinase-specific oncolytic Sendai virus has therapeutic efficacy in anaplastic thyroid carcinoma (ATC) mice models by induction of apoptosis. As a result, the Sendai virus could be used to treat ATC [95]. Another research examined how VSV wild type (wt) and M51R-mutant matrix protein (mMP) affected apoptosis, necroptosis, pyroptosis, and autophagy in esophageal squamous cell carcinoma (SCC). Their findings showed that VSV has an oncolytic function in tumor cells via apoptosis, necroptosis, and autophagy, but not pyroptosis [96]. Furthermore, Zhang and colleagues designed an oncolytic adenovirus that carried the TSLC1 (a tumor suppressor gene) and targeted the Wnt signaling pathway. Their findings reveal that recombinant adenovirus significantly reduces cancer-stem-like cell proliferation in HCC models through inducing apoptosis and autophagy [97].

Moreover, Parvoviruses, such as parvovirus H-1 (H-1PV), can attack and lyse cancer cells preferentially. Anti-cancer immunity is also induced by parvoviruses, which leads the immune system to kill tumor cells. The direct stimulation of apoptosis through parvoviral proteins NS1 is one of the proposed mechanisms of anti-cancer action [98].

### OVs and oxeiptosis

Oxeiptosis is a non-inflammatory, caspase-independent, ROS-sensitive and, an immune-silent form of cell death that is essential for protecting against inflammation produced by ROS or ROS-producing agents such as viral infections [99]. Influenza A virus leads to ROS production, which is detected by Kelch-like ECH-associated protein 1 (KEAP1). KEAP1 stimulates the transcription factor nuclear factor erythroid 2-related factor 2 (NRF2), which aids cell survival when ROS levels are low. Furthermore, KEAP1 binds to and deactivates the mitochondrial phosphatase PGAM5. At greater concentrations, KEAP1 loses contact with the phosphatase, triggering oxeiptosis [99, 100]. The cell is protected from more immunogenic types of death by participating in this form of cell death [99]. Furthermore, PGAM5 mutant mice have been observed to respond to influenza A virus infection with increased necrotic histology and quick death [99]. Through unharmed oxeiptotic signaling, a malignant tumor may preserve itself against ROS-induced immunogenic types of cell death, possibly reducing the efficiency of oncolytic viruses. Oxeiptotic cell death Downregulation in the TME appears to be a viable method for improving oncolytic virotherapy [101].

### OVs and pyroptosis

Pyroptotic cells, like apoptotic cells, utilize "eat-me" and "find-me" signals to promote macrophage phagocytosis, probably due to ATP release and phosphatidylserine (PS) exposure [102]. However, apoptotic cells release ATP less effectively than necrotic and pyroptotic cells [102]. Moreover, unlike apoptosis, pyroptosis is caused only by caspase-1 activity, which is initiated by the creation of a cytosolic complex known as the "inflammasome," resulting in extremely inflammatory consequences [20]. Pyroptosis may be induced by some OVs like herpes simplex virus type 2 (HSV-2) mutant, ΔPK [103]. In this context, Wang et al. discovered that NDV triggers the NLRP3 inflammasome, albeit the method by which inflammasome components detect NDV and whether this stimulation contributes to NDV's oncolytic properties remain unclear [104]. Also, Oncolytic HSV-1 RH2 was shown to release HMGB1, ATP and promote CALR translocation to the cell membrane, resulting in cell death with apoptosis and pyroptosis [105]. Overall, research on OV-induced pyroptosis and its specific processes and effects on cancer cells are still in their early phases, requiring additional research.

Destruction of tumor cells is mediated by OVs through two key processes: direct lysis of tumor-infected cells and indirect stimulation of host tumor-specific immunity [106]. OVs infect and proliferate in tumor cells, triggering lysis of tumor cells and the release of additional viral progeny which disseminate to cancer cells in the surrounding area. As a result, cancer cells treated with oncolytic viruses can initiate different cell death and release TAAs, DAMPs, and inflammatory cytokines, in order to restore the TME and provoke anti-tumor immunity [20, 52, 106].

## Effects of oncolytic viruses on the cancer-immune microenvironment

Tumor cells use various strategies to escape and inhibit anti-tumor immunity, leading to a "cold" immunosuppressive TME. TME contains tumor cells, blood vessels with endothelial cells, extracellular matrix (ECM), cancer-associated fibroblasts (CAFs), and a few infiltrating immune cells, such as regulatory T-cells (Tregs), myeloid-derived suppressor cells (MDSCs), and tumor-associated macrophages (TAMs). These immune cells have immunosuppressive features and, in collaboration with other cells in TME, generate and release growth factors, cytokines, and other chemicals that lead to the formation of an immunosuppressive TME [107].

Oncolytic virotherapy is developing as a successful strategy for restoring tumor immunosuppression [108]. OVs proliferate preferentially in tumor cells and destroy them by triggering ICD. Tumor cell lysis induced by OVs is correlated with the release of DAMPs, PAMPs, TAAs, and pro-inflammatory cytokines, all of which lead to the recruitment of immune cells in the TME and the DCs maturation, therefore stimulating anti-tumor immune responses. Generally, OVs appear to function in a multimodal manner, triggering ICD and powerful anti-tumor immunity [107, 109].

### OVs are stimulators of the immune system

To achieve the greatest anti-tumor impact before viral clearance, OVs must be engineered to proliferate and propagate rapidly inside tumors, but not in normal cells [110]. Several research on the genetic modification of OVs to selectively infect and eradicate tumor cells, as well as to improve anti-tumor immunity, have been undertaken [109, 111].

To promote viral propagation within the tumor cells, OVs may have anti-vascular effects and destroy the extracellular matrix. Also, within the microenvironment, OVs boost interactions between cytokine-induced killer cells, fibroblasts, and cancer cells, resulting in increased death of tumor cells [112]. Equipping viruses with immunomodulatory molecules like cytokines, which enhance the recruitment of immune cells to the tumor site, may augment anti-tumoral immune responses [113]. However, since viruses are identified as pathogens by the immune system, the ensuing antiviral response might represent a considerable obstacle for OVs [110]. For this, Li et al. revealed that repeated intratumorally delivery of the virus may boost the efficacy of anti-cancer treatment in a Syrian hamster model, providing a novel strategy to bypass antiviral immune response [110].

Tumor cells modify TME by producing high levels of VEGF, death ligands (PD-1, FasL, and TRAIL), anti-inflammatory cytokines, and several metabolites such as NO, RNS, and indoleamine 2 3-dioxygenase (IDO) [114]. These immunosuppressive agents not only decrease antitumor immunity, but also trigger stroma cells, and enhance tumor progression. Furthermore, Tregs, TAMs, and MDSCs can facilitate angiogenesis, tumor development, and metastasis by producing immunosuppressive agents including IDO, transforming growth factor-beta (TGF-β), ROS, arginase I, interleukin (IL)-10, and PD-L1 [115, 116].

On the other hand, killing local tumor cells may reverse the immunosuppressive features of the TME, allowing for enhanced TAAs release, cross-presentation to CD8^+^ T cells, and recruitment of anti-tumoral effector T cells [117]. All viruses trigger tumor cell lysis, resulting in the release of DAMPs, which stimulates phagocytosis and DC maturation [118]. DCs are recruited to the TME through ICD by binding HMGB1 and ATP to TLR4 and P2Y2, respectively. If dying tumor cells exhibit CALR, which binds to SR-A, LRP1, and SREC-1 on DCs, they will be phagocytosed quickly [9, 119] (Fig. 1). Also, binding HMGB1 to TLR-4 promotes cytokine production and cross-presentation of antigen. IFNs bind to IFNR and promote the production of a vast number of IFN-stimulated genes that help to induce adaptive immune responses. Mature DCs can present TAAs to cancer-specific T cells, resulting in anti-tumor immunity and cytolysis mediated by perforin and granzyme B [9].

According to research, tumor cells infected with Semliki Forest virus (SFV) elicited considerable T helper 1 (Th1)-cytokine production by DCs and triggered activation of antigen-specific T-cell [118]. Also, Feng-Ying Huang et al. demonstrated that NDV-MIP3 could produce humoral and cellular immunity and induce tumor lysis through ICD. Anti-tumor immune responses of NDV-MIP3(a recombinant oncolytic Newcastle virus expressing MIP-3α) were partly reliant on CD8^+^ T cells and partially dependent on CD4^+^ T cells [120]. Donnelly et al. revealed that the MVs improve innate immune response against tumors and MV-mediated cell death can stimulate adaptive immune responses against melanoma. Indeed, since inflammatory cytokines such I-IFNs and HMGB1 are released and stimulate DCs through MV-infected tumor cells, ICD occurs in human melanoma cells and enhances anti-tumor immune responses [121].

A number of viruses have progressed to the clinical stage in the treatment of cancer. For example, T-Vec, based on HSV-1, has been demonstrated to enhance tumor-specific CD8^+^ T cells while decreasing the number of Tregs. T-Vec has also been studied in a phase III trial in melanoma patients [66, 122], leading to FDA approval in 2015 for the melanoma patients as the first OV [66].

Since the stimulation of DCs is necessary for the activation of cytotoxic T cells, investigation on ICD has mostly concentrated on the DC–T cell axis. On the other hand, other effector cells probably have a function in ICD. Despite the fact that both NK and B cells play essential functions in anti-cancer responses, only NK cells have been studied in the field of ICD [9]. Tumor cell infection by various OVs induces the release of TAAs inside the TME, facilitating the detection of TAA-loaded cancer cells by CD4 + T cells [123]. In conclusion, the anticancer immune response induced by OV was revealed to overcome the immunosuppressive TME.

### OV-mediated induction of ICD

The potential of oncolytic immunotherapy to induce an anti-cancer immune response is dependent on ICD induction upon OV infection of tumor cells. Tumor cells may evade the immune system by altering their antigens and becoming undetectable to leukocytes, in a process known as immuno-editing. When OVs enter tumor cells, an inflammatory reaction is elicited, making the immune system more effective against virus-infected tumor cells. This is attributable to the fact that viruses can induce ICD [74].

Several studies have been undertaken in this area; for example, Takasu et al. examined the impact of oncolytic HSV-1 on DAMP production in squamous cell carcinoma (SCC) cells. They found that oncolytic HSV-1 RH2 induces SCC cells to produce DAMPs, which causes cell death. This immunogenic form of death may promote the potential of oncolytic HSV-1 to elicit anti-tumor immunity [105]. Furthermore, recent studies demonstrate that oncolytic NDV caused CALR exposure, HMGB1 and HSP70/90 release, as well as ATP secretion, resulting in ICD induction in melanoma cells [124]. Moreover, Wang et al. revealed that NDV/FMW, an oncolytic NDV strain FMW, triggered the production and exposure of various ICD markers in prostate cancer cells, including CALR, HSP70/90, and HMGB1. They also proposed that combining STAT3 inhibition with oncolytic NDV could enhance NDV-based anti-cancer actions in prostate cancer [125].

Interestingly, a lot of work has gone towards designing OVs which encode transgenes that trigger ICD in order to stimulate the immune system towards cancers [122, 126, 127]. For example, Zhu et al. have shown that the MV-Hu191 (Hu191 measles virus) strain is a suitable vector for foreign gene expression and can induce ICD, resulting in anti-tumor immune responses against nephroblastoma [128]. Also, Somma et al. claimed that using the adenovirus dl922-947, which has been designed to enable preferential propagation in tumor cells, might induce anti-tumor immune responses against Malignant pleural mesothelioma (MPM). They revealed that infection with dl922-947 had cytotoxic effects on MPM cell lines, influencing cell cycle progression, viability, and modulating ICD indicators such as HMGB1, ATP release, and calreticulin surface exposure [129].

Despite the OVs having the potential to induce tumor-specific immunity by stimulating T cells and NK cells through ICD, the immune system also can attack the OV by stimulating anti-viral pathways like type I IFN and neutralizing antibodies [130]. Therefore, the interaction between the immune system and OVs involves restrictive and stimulatory activities.

## CAR T cells characterization

CARs have an extracellular binding domain made up of a single-chain fragment variable (scFv) of antibody for recognition of HLA-independent antigen, also a transmembrane domain, and one or even more TCR intracellular signaling domains made up of a CD3 chain [131].

ScFv recognition domain allows CAR to bind to tumor cell-specific antigens. The initial concept connected scFv to an intracellular signaling component consisting of a part of the CD-3ζ chain to trigger activation of T cell upon antigen binding [132]. These two components are linked by a transmembrane domain and an extracellular hinge domain, leading in the simple form of CAR, known as a first-generation of CAR [133]. Soon after, attempts to enhance the existing CAR molecule resulted in the development of second and third-generation CAR structures that included signaling endodomains like CD28, 4-1BB (CD137), and inducible T cell co-stimulator (ICOS) in an effort to mimic the co-stimulation provided by APC during TCR recognition [134, 135]. Signaling domains from cytokine receptors or inducible production of inflammatory cytokines like IL-18 or IL-12 were introduced to fourth and fifth-generation CAR T-cells [136, 137].

## Immunogenic cancer therapies and efficacy of anti-tumor CAR T cell therapy

CAR T-cell therapy is a therapeutic T cell engineering approach, in which T lymphocytes obtained from patients are modified in vitro to display artificial receptors directed to a specific antigen of the tumor [138]. In fact, CAR T-cells enable T cells to bind specific antigens in the surface of tumor cells via an scFv recognition domain, resulting in HLA-independent tumor cell death. CAR T-cells establish an immune synapse, which is necessary for their cytotoxic activity. To their anti-tumor activities, these cells can use the Fas and Fas ligand axis, perforin and granzyme axis, and production of cytokines to sensitize of tumor stroma [133].

CAR T-cells have been extensively utilized in several hematologic malignancies in recent years, and owing to their efficacy in improving patient outcomes, the FDA approved them for lymphoma and leukemia [139]. In B cell malignancies such as non-Hodgkin lymphoma, acute lymphoblastic leukemia, and chronic lymphocytic leukemia, treatment with CD19-specific CAR T-cell demonstrated highly promising outcomes [140]. However, the usage of CAR T-cells in solid cancers has been met with some challenges, including CAR T-cell frailty in the immunosuppressive TME, restricted trafficking capacity, heterogeneity of tumor antigens, difficulty in identifying the ideal TAA target, and reduced proliferation and persistence of CAR T-cells in tumor site [141, 142]. The main goal of CAR-T immunotherapy is to alter T cells so that they can recognize and destroy cancer cells more effectively [138]. CAR T-cells would serve as a "living drug" against tumor cells after being administered to effectively treat a cancer patient. When CAR T-cells interact with their specific antigens on the tumor cell's surface, they attach to them, get triggered, and eventually destroy them [143].

To develop a great response, CAR-T cells must enter tumor cells, detect their relevant antigen, and fulfill their cytotoxic role in the TME, and then develop and persist as memory T cells that provide long-term immunity [144]. The poor results of CAR T cells in clinical studies of solid tumors suggested that monotherapy with CAR T cells is insufficient for the effective treatment of these malignancies, and that combination of them with other complementary treatments may be more helpful for cancer patient treatment [145].

## Combination of oncolytic virotherapy and CAR T cell therapy to maximize immunogenicity

The existence of three signals is required for anti-tumor T cell stimulation: a signal triggered by the T-cell receptor (TCR) interaction with antigen (Signal 1), a signal triggered by the interactions of the co-stimulatory molecules with cognate ligands upon APCs (Signal 2), and eventually, a signal triggered by the involvement of pro-inflammatory cytokines (Signal 3). The capacity to produce signals 1 and 2 is present in the second and third generations of CAR T-cells [146]. While ex vivo stimulation of CAR T-cells with cytokines can recapitulate signal 3 before delivery, and it may be further facilitated by altering the capacity of CAR T-cells to generate their cytokines and also to convey a series of cytokines sequentially as adoptive cellular immunotherapy [147–149]. The ability of OVs to induce I-IFN in the TME has recently been discovered. Also, the capacity of I-IFNs to activate Signal 3 in T cells has been discovered, so if OVs injected at the TME could increase the synthesis of this cytokine, it could be inferred that OVs have the ability to strengthen the cytotoxic activity of CAR T-cells on the TME; also, they could improve the safety of this therapeutic approach. Furthermore, I-IFNs have been shown to boost the cytolytic activity of T cells, increase clonal proliferation and also most critically, promote differentiation of T cells to memory cells [149, 150].

Overall, OVs play a key role in the first three phases of T cell therapy (Step 1: T cell priming, Step 2: trafficking and infiltration of T cells, and Step 3: circumventing immune suppression) [151]. The immunosuppressive TME is a major barrier to the utilization of CAR T immunotherapy in solid tumors. T cells that have entered the tumor must struggle with inhibitory factors and immunosuppressive cells like TAMs in the TME. Immunosuppressive cells may release powerful immunosuppressive mediators, including TGF, IL-10, arginase, and indoleamine 2,3-dioxygenase (IDO). OVs may overcome immunological suppression through eliciting robust, pro-inflammatory Th1 cell immune responses that significantly alter the TME [151]. However, despite this positive effect of OVs in altering immunosuppressive TME, it has become obvious that not all virus-induced outcomes are advantageous to CAR T, raising the issue of whether viruses operate as valets, directing CAR T to its active site, or vandals, triggering chaos and death in both tumor and T cells [13].

Tumor immune escape owing to loss of antigen is another challenge that CAR T-cells face. Target antigen availability on the tumor cell’s surface is crucial for CAR-T cell activation. On the other hand, Solid tumors are characterized by highly heterogeneous expression of antigen that may be totally absent. CAR-T cells are unable to detect antigen-negative tumor cells, allowing tumor development to proceed [152]. To address this, OVs may provide a suitable environment for T-cell growth and activity in malignant cells by selective lysis of tumor-infected cells and conveying danger signals. Another intriguing method is to employ CAR T-cells to transfer the OV to the tumor site [153]. The OVs loading on effector T cells could protect it against neutralizing antibodies and provide anti-cancer action following the viral release in the TME [154]. Transfer of OVs by CAR T-cells might improve the delivery of virus to the tumor site, and following oncolysis could recruit more CAR-T cells, forming a positive feedback loop [15].

**Fig. 2:**
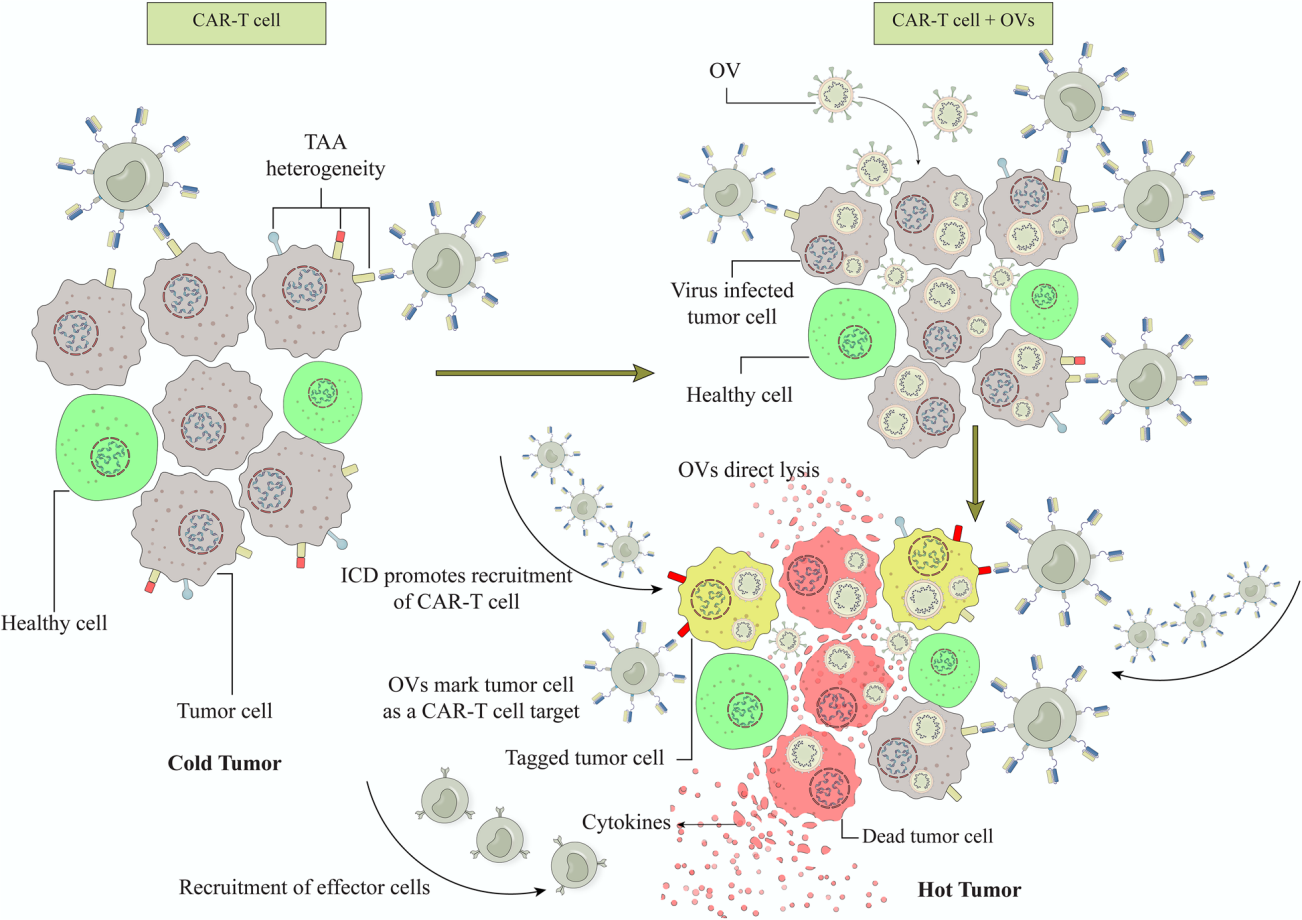
OVs could enhance the recruitment, activation, and expansion of CAR T cells by generating type I INFs and switching the tumor milieu from immunologically "cold" to a "hot" state. On the one hand, OVs cause tumors to die through immunogenic cell death (ICD), remove physical barriers, and send out a warning signal to T cells. OVs, on the other hand, can express the CD19 upon tumor cells as a specific target for CAR-T, enhancing CAR-T-mediated lysis. *OVs* oncolytic viruses, *CAR-T cell* Chimeric antigen receptor T cell, *ICD* Immunogenic cell death, *TAAs* tumor-associated antigens

In summary, it can be said that OVs could enhance the recruitment, activation, and expansion of CAR T cells by generating I-IFNs and switching the tumor milieu from immunologically "cold" to a "hot" state [14]. Moreover, recently a novel method has been developed which uses OVs as tumor-tagging to express specific antigen (like CD19) on tumor cells as a target for CAR T-cells (Fig. 2) [155].

Also, to circumvent depending on a tumor's native antigens, Aalipour et al. developed a thymidine kinase-disrupted VV for the specific delivery of CD19 to cancer cells. Furthermore, an in vitro investigation confirmed that CD19-CAR T-cells increased cytotoxic effect against two different cancer cell lines. Finally, they discovered that delivering CD19 to cancer cells could enhance CAR T-cells efficacy against tumor cells that displayed low amounts of antigen, indicating that it may be used to address antigen low evasion [156]. In another animal model, researchers designed a recombinant Ad encoding CD19 tag (AdC68-TMC-tCD19) that may be used to mark multiple solid cancers for recognition of anti-CD19-CAR T. As a result, this modified Ad could produce the generic tag and form immunological synapses between cancerous cells and CAR T-cells. Surprisingly, after injection of CAR T-cells, all of these tagged animals survived, and tumor progression was substantially suppressed by 92 percent in the premixed mouse model. They also constructed the replicative AdC68-Sur-E1A-TMC-tCD19 by combining the oncolytic capacity with tumor tagging. An oncolytic tagging method was shown to greatly increase mice survival and destroy existing tumors in mice models [157]. An oncolytic VV was modified to display the truncated CD19 (CD19t) molecule for selective delivery to tumor cells in a similar study by Park et al. Their findings showed that injecting OV19t into the tumor cells upregulate CD19t at the cell surface and facilitates tumor cell death after treatment with CD19-specific CAR T-cells [158].

Through the production of PAMPs and DAMPs (such as HMGB1) that operate on Toll-like receptors, an oncolytic viral infection of tumor cells causes ICD and an I-IFN response [159]. OV infection and following ICD of tumor cells have been shown to trigger innate and adaptive anti-tumor immune responses that promote effector function and T-cell migration within the TME [159]. Following OV-induced necrosis and pyroptosis of virus-infected tumor cells, tumor antigens attract Batf3 + DCs and scavenging macrophages, resulting in enhanced antigen presentation and subsequent stimulation of specific CD8^+^ and CD4^+^ T lymphocytes against the tumor antigen. These T cells can then traffic to tumor sites following the chemokine gradients produced by DCs in TME. CXCL-9 and CXCL-10 are secreted by DCs in TME, which recruit CD8^+^ T-cells, especially CAR T-cells, through CXCR3 [160].

Oncolytic virotherapy using modified Ad (OAd) may interrupt the TME by attacking tumor cells and adjacent stroma to increase the effectiveness of CAR-T cells, however, OAd delivery to solid tumors has proven challenging. In two different non-small cell lung cancer (NSCLC) models, researchers found that co-delivery of OAd and helper-dependent Ad (HDAd) expressing IL-12 and anti-programmed death-ligand 1 (PD-L1) by mesenchymal stromal cells may both directly destroy tumor spheroid formations in vitro and boost responses of CAR-T cells against orthotopic tumors in vivo. MSC-mediated systemic administration of a combinatorial Ad vector (Cad) boosted CAR-T cell recruitment and activation, as well as viral expression at the primary location of the tumor. Indeed, combining CAd MSCs with CAR-T cells promoted T cell penetration into tumors, improved effector cell activity, and increased production of cytolytic mediators IFN-γ, granzyme B, and perforin [161].

Various in vivo and in vitro studies have examined the possibility of synergistic effects of CAR T-cells and OVs, especially in solid tumors. For instance, the TGF-signaling pathway is thought to be important for establishing immunosuppression in TME. In this way, Li et al. investigated the efficacy of combining rAd.sT, an oncolytic Ad that targeted the TGF-signaling pathway, with CAR T-cell treatment in triple-negative breast cancer cells (MDA-MB231). Finally, they discovered that rAd.sT could destroy cancer cells and had significant anti-tumor activity in the initial stages, but the anti-tumor impact decreased as the stage progressed. Despite this, CAR T-cell immunotherapy demonstrated the strongest and longest-lasting tumor-specific response. Interestingly, CAR T combination with rAd.sT produced the highest anti-cancer immune responses and therapeutic outcomes [162].

Considering the significance of numerous cytokines, chemokines, and adhesion molecules in recruiting endogenous CTLs into the TME, it's possible that if an OV delivers these molecules, they could be attracted to tackle the obstacles of homing of CAR T-cell by improving their incursion to the tumor location [163]. Until now, a few preclinical and clinical investigations on the synergistic effects of cytokine-armed OVs and CAR T-cell treatment have been performed. For instance, Watanabe et al. employed an engineered adenoviral OV in pancreatic ductal adenocarcinoma (PDA) model that could generate either IL-2 or TNFα in combination with a mesothelin-directed 4-1BB-containing second-generation CAR. The generated IL-2 and TNFα both may inhibit the growth of cancer metastasis; hence the combination treatment was shown to increase the effectiveness of CAR T-cells. Moreover, the combination therapy was linked to macrophage M1 subset deviation, which enhanced DC maturation and local attraction of both transferred donor CAR T-cells and non-CAR host T cells via the TNF-inducible chemokine secretion like CCL-2, CCL-5, and CXCL-10. Notably, Chmielewski and Abken demonstrated in both metastatic lung adenocarcinoma and pancreatic carcinoma mouse models that IL-18-secreting CAR T-cells can stimulate a high T-bet and low levels of FoxO1 in CAR T-cells and enhance tumor penetration of NKG2D^+^ NK cells while decreasing the rate of suppressive macrophages and Tregs [164].

Also, in the syngeneic mouse mesothelioma model, direct intratumoral delivery of a CXCL-11 armed vaccinia OV strain resulted in an elevation in anti-tumor cytotoxic T cell infiltration [165]. Furthermore, this model revealed considerable immune suppressive cytokines and chemokines downregulation such as CCL-22 (a Treg chemoattractant), TGF, and Cyclooxygenase-2 (COX2), as well as perforin and granzyme B overexpression simultaneously [165]. In the second investigation, Moon et al. assessed the synergistic activity of CXCL-11 and mesothelin-redirected CAR T-cells on patients with mesothelioma and a murine model. CXCL11 was transmitted to the specific tumor tissue in this investigation either by subcutaneous administration of a VV equipped with CXCL-11 (VV.CXCL-11) or by overexpression in transferred T cells adoptively transfected with a lentiviral transgene cassette that produced a 4-1BB carrying both anti-mesothelin CAR T-cell and CXCL-11 [166]. Nevertheless, although both techniques were demonstrated to be capable of increasing the expression of CXCL-11 in TME, only VV.CXCL11 was able to improve anti-tumor activity after adoptively transferring T cells utilizing mesothelin-redirected CARs [166]. Nishio et al. Showed that equipping an oncolytic Ad (Ad524) with the chemokine RANTES and the cytokine IL15 could improve the migration and survival of CAR T-cells and suppress the growth of neuroblastoma in mice [167]. Also, the combination of RANTES and IL-15 was later shown in similar research to boost T-cell trafficking to tumor locations as well as generate a suitable environment inside the TME to promote the persistence of immune cells [168].

Recently, Huang et al. developed an IL-7-loaded oncolytic Ad (oAD-IL7) and combined it with B7-H3-specific CAR T-cells to treat orthotopically glioblastoma-grafted mice. They showed that combining oAD-IL7 with CAR T-cells contributed to the increased proliferation of T cells and decreased apoptosis of T cells in vitro, as well as longer survival and lower tumor burden in vivo. Indeed, this research illustrated that oAD-IL7 is a potential supplementary treatment for enhancing the therapeutic effectiveness of B7H3-CAR-T in glioblastoma through establishing stimulating signals for T cells that infiltrate to tumor site [169]. Moreover, in another study to enhance CAR T-cells activity in a mouse model of head and neck squamous cell carcinoma, oncolytic Ad was equipped with the IL-12 (an immune-stimulatory cytokine) and a PDL1-blocking antibody, combined with HER2/neu (human epidermal growth factor receptor 2)-specific CAR T-cells for the treatment of head and neck cancer. Finally, it was discovered that CAd12-PDL1 enhances the anti-tumor actions of HER2.CAR T cells, hence inhibiting the growth of primary and metastatic cancers [170]. Furthermore, therapeutic transgenes can be expressed selectively in the TME by genetically engineering OAds. In this regard, Tanoue et al. developed a novel method for prostate cancer immunotherapy that included an oncolytic Ad (Onc.Ad), HDAd expressing a PD-L1 blocking mini-antibody, and HER2.CAR T-cells. Their findings showed that this combination therapy improved anti-tumor immunity when compared to HER2.CAR T-cells therapy alone or HER2.CAR T-cells plus Onc.Ad, as well as the advantages of PD-L1 mini-body created locally, outperform anti-PD-L1 immunoglobulin (Ig)G infused [171].

Interestingly, combining OVs with some modified compounds to engage the TCR complex of T cells has revolutionized the potential of OV compounds to restore the suppressive TME and promote the increased tumor-specific immune response. BiTEs are bispecific monoclonal antibodies composed of two linked single-chain variable fragment (ScFv) antibody domains (anti-CD3 fused to an anti-TAA). OVs could secrete BiTEs, which cause tumor cell death. These compounds were engineered to bind with both CD3 from the TCR complex and TAA on tumor cells simultaneously [172]. Although BiTEs can infect and proliferate in tumor cells, normal cells can resist OV infection. Expression of TAAs induced by OVs works in combination with BiTE and CAR T-cell therapies [148].

Various research has investigated the potential synergy between OVs and BiTEs. In this way, Wang et al. developed a new T-cell engager armed VV (TEA-VVs) with the capacity to produce bispecific antibodies that engage with either EphA2 (an antigen in cell surface) or CD3. As a result, OV infection might trigger non-infected tumor cells to be eradicated by T cells. Furthermore, when this oncolytic therapy is combined with a HER2-redirected CAR, the decrease in the survival of triple-positive HER2/ EphA2/ A549 tumor cells is accelerated, indicating that this method is effective in addressing the heterogeneity of cold tumors and preventing CAR-mediated antigen evasion [173]. The efficiency of combination therapy was further strengthened by the previous research findings that showed the capacity of TEA-VV to proliferate in HER2- redirected CAR T-cells, indicating that CAR might be employed as a safe delivery vehicle for TEA-VV, protecting it from host exclusion [174] This unique technique might be used to treat tumor stroma and any other situation where specific T cell immune responses are restricted owing to immunosuppressive circumstances or physical limitations. In a xenograft mouse model of melanoma, Yu et al. investigated the possibility of TEA-VV encoding BiTE targeting murine CD3 and fibroblast activation protein (FAP). This in vitro investigation found that mFAP-TEA-VV may drive bystander elimination of noninfected FAP + stromal cells in the presence of murine T cells. Furthermore, in vivo transfection of mFAP-TEA-VV led to increased viral titers and decreased metastatic cancer burden [175].

Porter et al. recently published a study that showed increased potency, breadth, and duration of anti-cancer function of CAR T-cells employing cytokine-expressing OV and BiTE-checkpoint blockage. In this investigation, CD44 variant 6 (CD44v6) specific BiTE was exposed to CAdDuo, a binary Ad capable of producing IL-12 and PD-L1Ab, to create CAdTrio [176]. Additionally, CD44v6 BiTE has been shown to improve the susceptibility of CD44v6-expressing cancer cell lines to the cytotoxic impact of HER2-specific CAR T-cells. Also, in orthotopic HER2^−/−^ CD44v6^+^ and HER2^+^ cancers, CD44v6 BiTE was observed to enhance the anti-cancer function of HER2-specific CAR T-cells [176]. In this regard, recently Shaw A.R et al. used an oncolytic adeno-therapy which generates cytokine, immune checkpoint inhibition, and a protective switch (CAdTrio) to boost the potency, breadth, and duration of anti-PDAC HER2-specific CAR T-cell (HER2.CART) function. Eventually, they showed that CAdTrio and HER2.CARTs work together to remove metastatic pancreatic adenocarcinoma (PDAC), and that they might be a potential combination therapy for patients with PDAC [177].

At a low density of antigen, EGFR.BiTE-armed Ad (OAd-BiTE) was shown to boost CAR T-cell anti-tumor activity. Furthermore, it was proposed that using the second-generation ICOS-armed anti-FRα CAR in combination with OAd-BiTE could perhaps bring some promising outcomes after it was discovered that heterogeneous expression of FRα could lead to the resistance inducing against single CAR T cells therapy in NSG mice with SKOV tumors [178].

Recently, Altomonte et al. demonstrated that combining CAR T-cells with fusogenic VSV-NDV improved CAR T-cell treatment in the melanoma immunocompetent mouse models. They discovered that by increasing MHC-I expression and keeping low PD-L1 expression levels on cancer cells, this combination treatment had favorable impacts on the suppressive features of TME. Indeed, this combinatorial method in vitro and in vivo resulted in synergistic cytotoxic activity as well as increased T cell attraction to the site of virus-infected cancer cells [179]. The hybrid VSV-NDV platform was reported as a chimeric OV capable of stimulating the immune system, with much superior safety and efficacy compared to VSV after treatment response to a mouse hepatocellular carcinoma model [180]. Also, Wenthe et al. evaluated the therapeutic efficacy of Ad expressing 4-1BBL and CD40L (LOAD703) in combination with CAR T-cell. They discovered that LOAD703 can trigger cell lines of B cell lymphoma to enhance the expression of surface T cell co-stimulatory molecules. Furthermore, lymphoma cells infected with LOAd703 increased the release of various chemokines (CCL3, CCL4, CXCL10,) that are important for homing of immune cells, resulting in increased migration of CAR T-cells. To summarize, the treatment with immune-stimulatory LOAd703 is a promising method for inducing anti-tumor immune cells and enhancing CAR T-cell in B-cell lymphoma therapy [181].

These data show that, while solid tumors are programmed to avoid immunotherapies, combining OVs and CAR T-cell immunotherapy may overcome these escape mechanisms.

## Hurdles of combination therapy with oncolytic viruses and CAR T cells

As there are so many OVs, predicting which one will perform better in synergy with CAR T-cells is complicated. Also, while the virus's potential to attract effector T-cells to the tumor site is well established, developing optimal delivery methods and dosing regimens remains challenging [182, 183]. Intratumoral delivery of the OV leads to higher levels of virus in the infused cancers, but it is difficult to modify the immunosuppressive milieu in visceral tumors or metastases, and non-injected cancer lesions are far less likely to acquire any virus. Although systemic intravenous delivery is simpler to distribute and may be effective in reaching all metastasis sites, successful viral neutralization in the circulation, particularly with a large amount of neutralizing antibodies developed after the first administration of the virus, will create a hurdle to repeated administration. As a result, finding techniques to protect given viral preparations against antibody inactivation seems to be a high priority [15, 184].

It is also necessary to establish the order in which the OV and CAR T-cells are administered. The virus should theoretically be administered first to switch the tumor's immunosuppressive microenvironment, followed by a direct lytic impact on infected tumor cells and the establishment of a more suitable environment for the recruitment of CAR-T cells. Notably, neoantigens produced by OVs during tumor cell lysis are much less immunogenic than viral antigens [185–187]. To improve epitope spread, new approaches are required to boost the immunogenicity of tumor antigens while decreasing the immunodominance of viral epitopes [188].

Moreover, a recent study showed that OV-associated I-IFN has a detrimental effect on CAR T-cell survival. Furthermore, a recent study found that OV-associated I-IFN has a negative effect on CAR T-cell survival, hence rendering CAR T-cells unresponsive to I-IFN, which enhances combination therapy [189].

## Conclusion and outlook

To date, studies on solid tumors using a combination of CAR T-cell therapy and OV-based immunotherapy have shown a synergistic impact, addressing the fundamental drawbacks of each monotherapy separately. The options for combining multiple OVs with anti-tumor CAR T-cells are almost endless, and the broad usage of this approach offers hope for enhancing solid tumor therapy. Inflammation generated by OVs must be considered as a multi-component event that might either be advantageous or detrimental to the development of anti-tumor immunity. Therefore, caution must be exercised when using treatments that target tumor inflammation, and such combination strategies should be tested in immunocompetent models with no restrictions on the cross-reactivity between CAR T-cells and their surroundings.

The capacity to further modify both CAR T-cells and OVs with customized transgenes extends the range of their combined usage and foreshadows a time when a virtually infinite number of viral products and patient-specific bespoke cellular are routinely delivered to cancer patients. Employing OVs to enhance ICD holds a lot of promise for reactivating tumor-specific immune responses in patients with cancer. This feature could provide a cancer therapy that is relatively cost-effective, short-term, and individualized. Finally, OVs can be efficiently combined with CAR T-cells, assisting in overcoming crucial hurdles in the battle against cancer by providing synergistic effects. Although many concerns must be addressed to completely realize the therapeutic effect of OVs, oncolytic virotherapy will undoubtedly become a prominent part of future cancer treatments.

## Data Availability

Not applicable.
